# Single-cell transcriptome analysis reveals the clinical implications of myeloid-derived suppressor cells in head and neck squamous cell carcinoma

**DOI:** 10.3389/pore.2023.1611210

**Published:** 2023-07-05

**Authors:** Wenru Jiang, Kangyao Hu, Xiaofei Liu, Jili Gao, Liping Zhu

**Affiliations:** ^1^ Department of Implant and Prosthodontics, The First Affiliated Hospital of Harbin Medical University, Harbin, China; ^2^ Department of Implant and Prosthodontics, Harbin First Hospital, Harbin, China

**Keywords:** head and neck squamous cell carcinoma, myeloid-derived suppressor cell, single cell RNA sequencing (scRNA-seq), prognosis, immunotherapeutic

## Abstract

Head and neck squamous cell carcinoma (HNSC) is the most common malignant tumor that arises in the epithelium of the head and neck regions. Myeloid-derived suppressor cells (MDSCs) are one of the tumor-infiltrating immune cell populations, which play a powerful role in inhibiting anti-tumor immune response. Herein, we employed a single-cell RNA sequencing (scRNA-seq) dataset to dissect the heterogeneity of myeloid cells. We found that *SPP1* ^+^ tumor-associated macrophages (TAMs) and MDSCs were the most abundant myeloid cells in the microenvironment. By cell cluster deconvolution from bulk RNA-seq datasets of larger patient groups, we observed that highly-infiltrated MDSC was a poor prognostic marker for patients’ overall survival (OS) probabilities. To better apply the MDSC OS prediction values, we identified a set of six MDSC-related genes (*ALDOA*, *CD52*, *FTH1*, *RTN4*, *SLC2A3*, and *TNFAIP6*) as the prognostic signature. In both training and test cohorts, MDSC-related prognostic signature showed a promising value for predicting patients’ prognosis outcomes. Further parsing the ligand-receptor pairs of intercellular communications by CellChat, we found that MDSCs could frequently interact with cytotoxic *CD8* ^+^ T cells, *SPP1* ^+^ TAMs, and endothelial cells. These interactions likely contributed to the establishment of an immunosuppressive microenvironment and the promotion of tumor angiogenesis. Our findings suggest that targeting MDSCs may serve as an alternative and promising target for the immunotherapy of HNSC.

## Introduction

Head and neck squamous cell carcinoma (HNSC) is the sixth most common form of malignant tumor occurring in the epithelial tissues of the head and neck regions [1–3]. Despite the advancement in treatment approaches, such as surgical resection and multidisciplinary treatments involving radiotherapy and chemotherapy, the overall 5-year survival rate remains below 70%, particularly for patients in advanced stages of the disease [2–4]. Hence, comprehending the tumor microenvironment of HNSC, identifying therapeutic targets, and formulating novel treatment approaches are imperative.

Myeloid-derived suppressor cells (MDSCs) are a heterogeneous population of pathologically activated neutrophils and monocytes that accumulate in the tumor microenvironment and play a critical role in promoting tumor growth and immune evasion [5]. MDSCs have been identified in various types of cancer, including lung cancer, breast cancer, melanoma, and others [6–8]. MDSCs promote tumor growth by suppressing the immune response, particularly T cell activation, and function, which are critical for controlling cancer growth [9, 10]. Immunotherapies that target MDSCs include monoclonal antibodies and small molecule inhibitors. These therapies work by blocking the recruitment of MDSCs to the tumor microenvironment, inhibiting their function, and promoting T cell activation [11–13]. A recent clinical study revealed that the administration of tadalafil, a phosphodiesterase-5 (PDE5) inhibitor, in HNSC patients resulted in a decrease in circulating MDSCs [14, 15]. Moreover, the treated patients exhibited reduced expression of iNOS and arginase in these cells, along with an increased presence of spontaneously generated tumor-specific T cells [14, 15]. Notably, in a co-culture system, HNSC cells had the potential to induce MDSCs differentiation from peripheral blood mononuclear cells and upregulate the expression of iNOS and arginase [16], which further indicated that immunotherapy strategies targeting MDSCs hold great promise.

In this study, we utilized a public single-cell RNA sequencing (scRNA-seq) dataset to investigate the heterogeneity of myeloid cells in HNSC. Our analysis revealed that *SPP1* ^+^ tumor-associated macrophages (TAMs) and MDSCs were the most abundant myeloid cells in the tumor microenvironment. We also discovered that highly infiltrated MDSCs were associated with poorer overall survival rates in HNSC patients. To further explore the potential clinical application of MDSCs as a prognostic marker, we identified a set of six MDSC-related genes that could be used as a prognostic signature. Using this signature, we were able to predict patients’ prognosis outcomes with promising accuracy in both training and test cohorts. By examining intercellular communications, we found that MDSCs were able to suppress the activity of cytotoxic *CD8* ^+^ T cells and recruit *SPP1* ^+^ TAMs to shape an immunosuppressive microenvironment that promoted tumor angiogenesis. Overall, our findings suggest that targeting MDSCs may provide a promising therapeutic strategy for the immunotherapy of HNSC.

## Materials and methods

### Data collection

Single-cell dataset from Peng et al. was employed in our study, containing six tumor tissues of head and neck cancer (HNSC) patients (GSE172577) [17]. Bulk gene expression profiles of HNSC were downloaded from The Cancer Genome Atlas (TCGA) program. We also obtained the clinical information of corresponding samples of TCGA. In addition, two independent datasets GSE65858 [18] and GSE41613 [19] were employed as the test cohorts, which included 270 and 97 samples, respectively. The patient’s clinicopathological information is listed in Supplementary Table S1.

### scRNA-seq data processing, batch correction, and clustering

We imported the unique molecular identifier (UMI) count data generated by 10x genomics into Seurat (V4.1.0) [20]. To remove the low-quality cells, we filtered (1) the cells with more than 20% mitochondrial counts; (2) cells expressing lower than 300 genes or more than 4,000 genes (Supplementary Figure S1). We also employed Scrublet with the default parameters to identify putative doublets [21]. The remaining 45,876 cells from six patients were normalized, scaled, and then used for batch correction. We took the Seurat-v3 batch correction strategy, anchors across patients were identified using the function *FindIntegrationAnchors,* and the data were finally integrated using the “*IntegrateData*” function. The assay “integrated” was used for downstream analysis. We next used the “*FindVariableFeatures*” function to choose the top 2000 highly variable genes based on the “vst” selection method. Principal component analysis (PCA) was performed and the top 30 PCA components were used for Uniform Manifold Approximation and Projection (UMAP) [17, 22–24]. Subsequently, the cells were clustered on UMAP space using the Lovain algorithm on the k-nearest neighbors graph constructed using gene expression data as implemented in *FindNeighbors* and *FindClusters*. We further annotated major cell types according to the gene expression of well-known markers: T/NK cells (*PTPRC*, CD3D), mast cells (*TPSAB1*, *TPSB2*), B cells (*CD79A*, *MS4A1*), myeloid cells (*C1QA*, *C1QB*), fibroblasts (*LUM*, *DCN*), endothelial cells (*PECAM1*), keratinocytes (*KRT15*, *KRT19*), epithelial cells (*KRT13, KRT14, EPCAM*), and proliferating cells (*TOP2A*, *MKI67*).

### Identification of myeloid subpopulations

We next subgrouped the myeloid cells. Based on the UMAP space, we applied the Lovain algorithm on the k-nearest neighbors graph using the function of *FindNeighbors* and *FindClusters* in the “Seurat” package. Markers were identified using the Wilcoxon Rank Sum test in each myeloid subgroup using the function *FindAllMarkers*.

### Cell-type deconvolution

We employed the BayesPrism algorithm to deconvolute the infiltration levels of myeloid cells [25]. BayesPrism is a statistical framework for cell-type deconvolution, which is the process of inferring the proportion of cell types present in a heterogeneous mixture of cells based on gene expression data. The analysis was performed using the default parameters of the R package “BayesPrism” (https://github.com/Danko-Lab/BayesPrism).

### CopyKAT analysis

CopyKAT [26] uses integrative Bayesian approaches to find genome-wide aneuploidy at 5MB resolution, and cells with large genome-wide aneuploidy were identified as tumor cells (Supplementary Figure S2). UMI count matrix was used as input, and others were default parameters (id.type = “S”, ngene.chr = 5, win.size = 25, and KS.cut = 0.1).

### CytoTRACE analysis

We utilized the CytoTRACE [27] to compare differentiation states among HNSC tumor cells (https://cytotrace.stanford.edu/). CytoTRACE analyzes the number of uniquely expressed genes per cell, as well as other factors like distribution of mRNA content and the number of RNA copies per gene, to calculate a score assessing the differentiation and developmental potential of each cell (lowest differentiation and highest developmental potential at 1; highest differentiation and lowest developmental potential at 0). CytoTRACE analysis was conducted using default parameters. In addition, we evaluated the activities of cancer hallmark pathways from MSigdb (https://www.gsea-msigdb.org/gsea/) using the R package AUCell [28] with default parameters. Subsequently, Spearman’s correlation was calculated between the hallmark activity and CytoTRACE score.

### Construction of the MDSC-related prognostic signature

We first divided the TCGA-HNSC cancer samples into three parts (two parts as the “training” set and one part as the “test” set) to apply 3-fold cross-validation. Then, we applied the univariate Cox regression model to screen the MDSC-related genes (log_2_FC > 0.25 and expression percentage >25%) that were associated with patients’ overall survival (OS) in the training set of the TCGA-HNSC cohort. Genes with *p*-value <0.05 were identified as the candidate prognosis-related genes (Supplementary Table S2). Afterward, we used a stepwise multivariate Cox regression model based on the Akaike information criterion (AIC) value to analyze the candidate MDSC-related genes and selected the ones that minimized AIC to achieve the best model fit [29]. We subsequently calculated the risk score [30] for each patient by the linear combination of expression values weighted by the coefficient from the multivariate Cox regression model, $risk\;score=\sum_{i}^{n=6}{coef}_{i}*\exp_{i}$ , where i represented the i^th^ MDSC-related gene, exp denotes the expression levels of MDSC-related genes. We used the median value of patients’ risk scores to determine the high-risk and low-risk groups. Kaplan-Meier (KM) analysis with log-rank test was applied to compare the survival difference between patients’ risk groups using the R package “survival.”

### CellChat analysis

We employed the CellChat computational tool to analyze communication among microenvironment cells [31]. CellChat uses a scoring system to identify the most likely interactions between cells based on the expression of genes encoding ligands and receptors.

In brief, we followed the official workflow and imported gene expression data using the function of *createCellChat*. We then applied the functions of *identifyOverExpressedGenes*, *identifyOverExpressedInteractions*, *projectData* to detect significant cell-cell interactions among the investigated cells. The analysis was conducted by the R package CellChat (https://github.com/sqjin/CellChat).

## Results

### The single-cell landscape of HNSC tumor microenvironment

**FIGURE 1:**
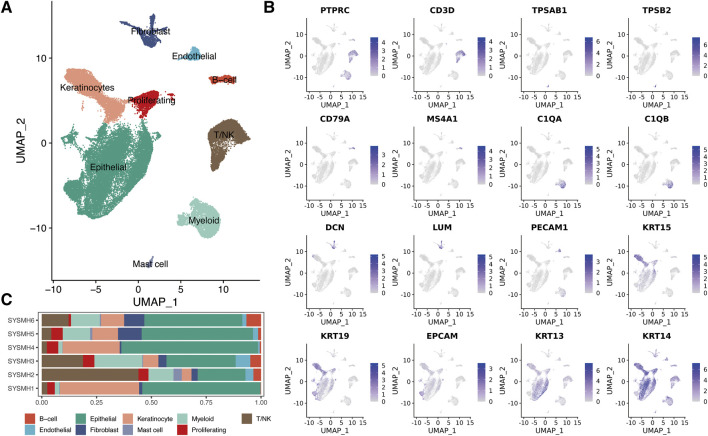
A global overview of TME in HNSC at single-cell resolution. **(A)** UMAP plot of major cell populations, dots colored by different cell types. **(B)** The expression level of cell type specific gene markers. **(C)** Bar plot showing the sample type fractions relative to the total cell count per cell type.

To dissect the landscape of the tumor microenvironment of head and neck squamous cell carcinoma (HNSC), we obtained scRNA-seq data from 6 untreated HNSC primary patients (GSE172577) [17]. A total of 45,876 passed the initial quality control and were retained for downstream analysis (Supplementary Figures S1A,B). We merged all scRNA-seq data and performed gene expression normalization, scaling, dimension reduction, batch correction, and cell clustering to identify coarse cell types (Materials and methods). Nine major cell types were detected based on the gene expression of canonical cell markers, including epithelial cells, keratinocytes, fibroblasts, endothelial cells, myeloid cells, T/NK cells, mast cells, B cells, and proliferating cells (Figures 1A, B). The proportions of these major cell types exhibited significant variation among the different patients (Figure 1C). Notably, compared to epithelial cells, tumor-infiltrating immune cells (myeloid cells, T/NK cells, mast cells, and B cells) showed lower sample heterogeneity (Supplementary Figures S1C).

### Dissecting the heterogeneity of myeloid cells

**FIGURE 2:**
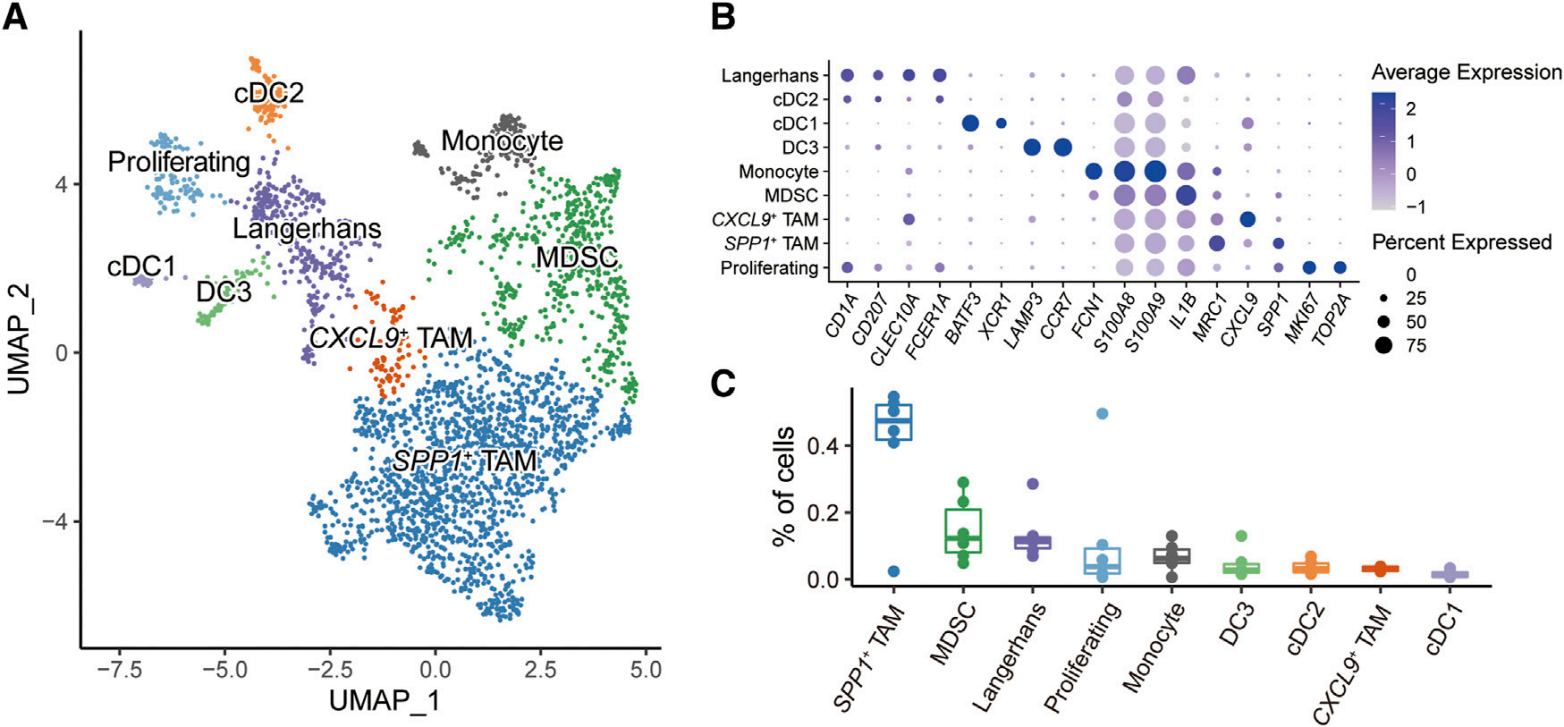
Subpopulations of myeloid cells in HNSC microenvironment. **(A)** UMAP plot of myeloid subpopulations, dots colored by different cell types. **(B)** Dot plot showing the expression level of cell-type-specific gene markers. **(C)** Box plot showing the fractions of cells in each sample.

Myeloid cells play critical roles in regulating the immune response in the HNSC tumor microenvironment [32, 33]. Overall, 3,026 myeloid cells were further reclassified into nine populations (Figure 2A). Known representative genes were used to recognize cell identities (Figure 2B), including three subtypes of dendritic cells (DCs): cDC1 (*BATE3*, *XCR1*), cDC2 (*CLEC10A*, *FCER1A*), DC3 (*LAMP3*, *CCR7*); two types of tumor-associated macrophages (TAMs): *SPP1* ^+^ TAM (*SPP1*, *MRC1*), *CXCL9* ^+^ TAM (*CXCL9*); monocytes (*FCN1*); myeloid-derived suppressor cells (MDSCs) (*S100A8, S100A9, IL1B*); Langerhans cells (*CD1A*, *CD207*); proliferating cells (*MKI67*, *TOP2A*). We next investigated the proportions of myeloid subpopulations among HNSC patients (Figure 2C). The result showed that *SPP1* ^+^ TAM and MDSC had higher fractions compared to other myeloid cells. Notably, both *SPP1* ^+^ TAM and MDSC played a critical role in shaping the immunosuppressive tumor microenvironment [34–37].

### Highly-infiltrated MDSCs prompt poor prognostic risks

**FIGURE 3:**
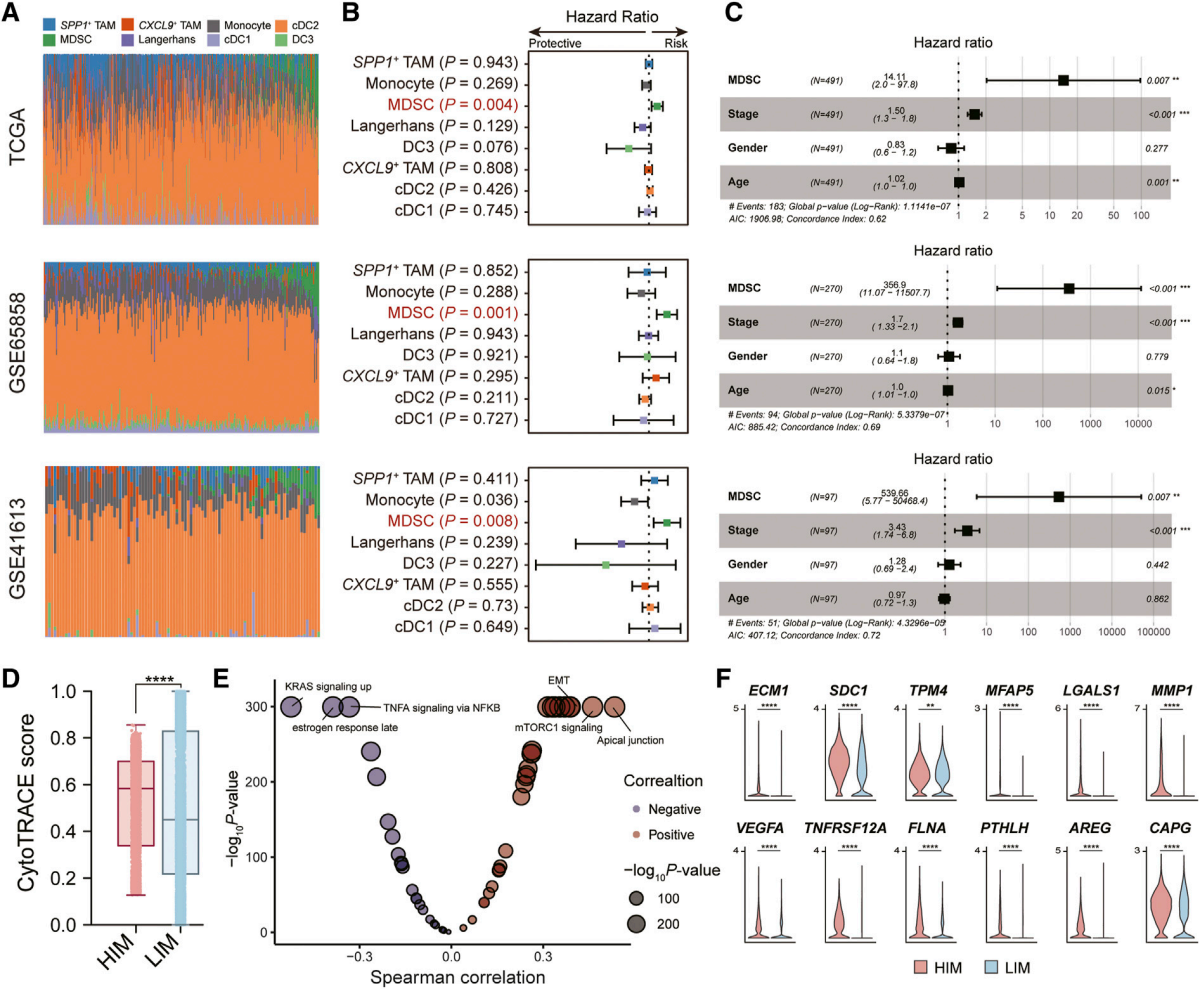
Tumor-infiltrated MDSC was associated with patients’ prognostic outcomes. **(A)** Bar plot showing the infiltration levels of myeloid subpopulations evaluated by the BayesPrism algorithm. **(B)** Forest plot showing the result of univariate Cox-regression analysis for correlation between the myeloid cell infiltration levels and the overall survival. **(C)** Forest plot showing the result of multivariate Cox-regression analysis of the MDSC infiltration levels and the overall survival adjusting the effects of tumor stage, age, and gender. Three independent datasets were used: TCGA cohort (*n* = 491) (top), GSE65858 (*n* = 270) (middle), and GSE41613 (*n* = 97) (bottom), respectively. **(D)** Boxplot depicting the comparison of CytoTRACE scores between tumor cells in HIM [high-infiltrated MDSC tumor microenvironment (TME)] group and those in LIM [low-infiltrated MDSC TME] group. *p*-value was calculated by the Kruskal-Wallis test. **(E)** Volcano plot showing the correlation between CytoTRACE score and the activity of cancer hallmark pathway. **(F)** Violin plot showing the expression levels of EMT-related genes in tumor cells in high/low-infiltrated MDSC TME. *p < 0.05, **p < 0.01, ***p < 0.001, and ****p < 0.0001, as calculated by Mann Whitney U test.

To understand the role of myeloid cells in HNSC patients, we performed the deconvolution analysis using the BayesPrism algorithm [25]. We evaluated the infiltration levels of myeloid cells in three independent bulk RNA-seq datasets (TCGA, GSE65858 [18], and GSE41613 [19]) (Figure 3A). By constructing the univariate Cox regression model, we found only the infiltration levels of MDSC showed a significant association with the patient’s overall survival (OS) probability in all three datasets (Figure 3B). Patients with highly-infiltrated MDSCs usually suffer poor prognostic outcomes, suggesting MDSC infiltration was a prognostic risk factor (HR > 1 and *p*-value <0.05). In addition, we constructed the multivariate Cox regression model to explore the correlation between MDSC infiltration and patient OS probability adjusting the effects of tumor stage, age, and gender. The result showed that the MDSC infiltration was an independent factor for predicting the prognostic risk (Figure 3C).

We next explored the impact of MDSC infiltration on tumor cells in the tumor microenvironment (TME). Based on the median levels of MDSC infiltration, we divided the high-infiltrated-MDSC TME (HIM) (patients SYSMH2, SYSMH3, SYSMH5) and low-infiltrated-MDSC TME (LIM) (patients SYSMH1, SYSMH4, and SYSMH6). We then used CopyKAT [26] to infer copy number alterations at 5 Mb resolution by averaging large chromosomal regions (1 Mbp) and identified the HNSC tumor cells in each sample (Supplementary Figure S2). By applying the CytoTRACE algorithm to evaluate tumor cell differentiation states [27], we found that tumor cells in HIM TME had higher developmental potential than those in LIM TME (Figure 3D). Further correlation analysis indicated that apical junctions, mTORC1 signaling, and epithelial-mesenchymal transition (EMT) were the most associated processes with tumor cell differentiation (Figure 3E) (See the ‘Methods’ section). Specifically, we also found some EMT-related genes were more highly expressed in the HIM tumor cells than in LIM tumor cells (Figure 3F). These results suggested that MDSC infiltration might play an important role in tumor cell development.

### An MDSC-related gene signature shows robust prognostic predictive values

**FIGURE 4:**
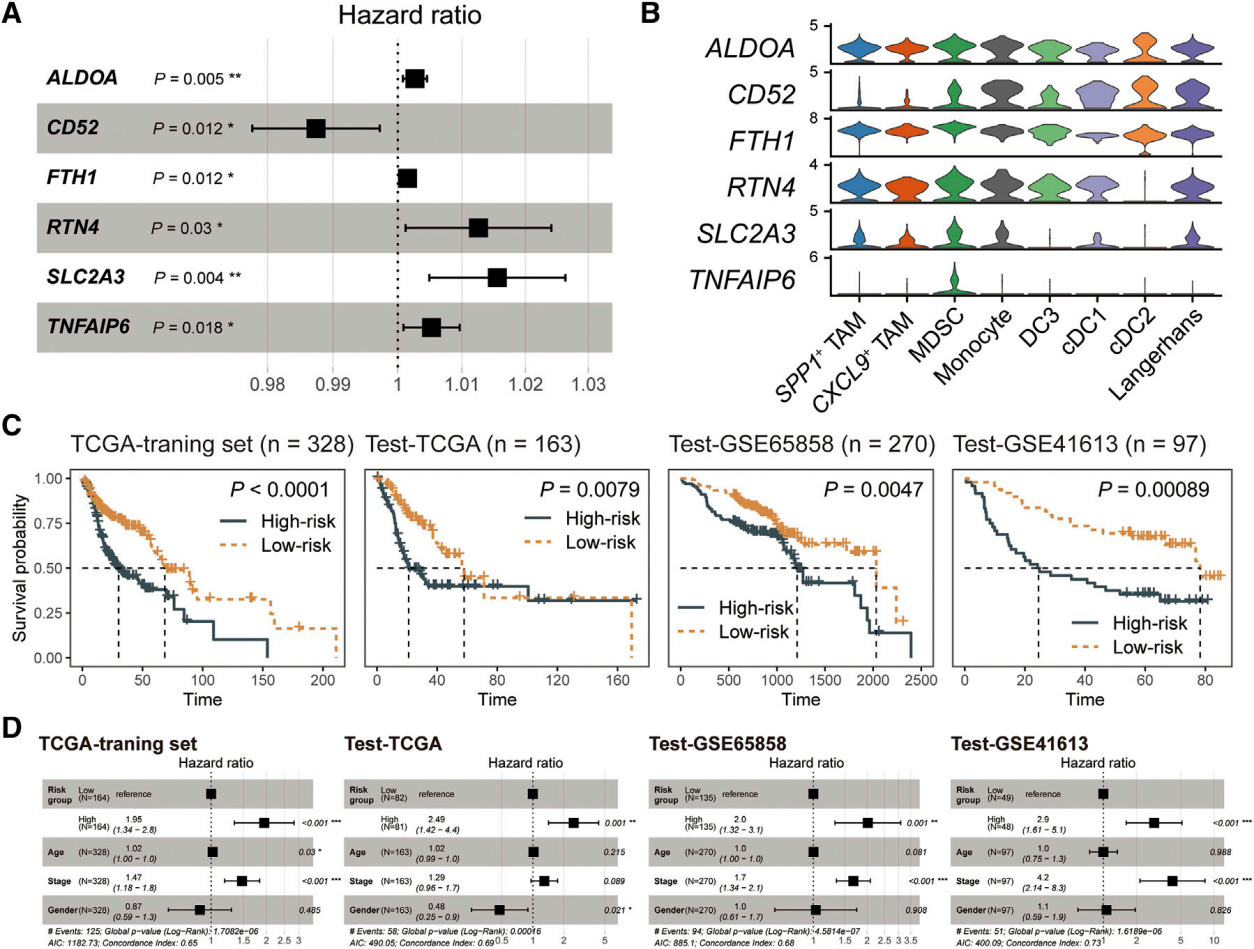
The MDSC-related prognostic signature of HNSC. **(A)** Forest plot showing the result of multivariate Cox-regression analysis for correlation between the MDSC-related genes and the overall survival. **(B)** Violin plot showing the expression levels of the MDSC-related prognostic signature across myeloid subpopulations. **(C)** Kaplan-Meier curve of HNSC samples stratified by the risk groups with log-rank test *p*-value provided. **(D)** Forest plot showing the result of multivariate Cox-regression analysis for correlation between the risk group and the overall survival by adjusting the effects of gender, age, and tumor stages.

To better apply MDSC in survival prediction, we explored the prognosis values of MDSC-related genes (log_2_FC > 0.25 and expression percentage >25%). We divided the TCGA-HNSC cancer samples into three parts, where the TCGA training set had twice as many patients as the TCGA test set, and constructed the univariate Cox regression model based on the expression levels of MDSC-related genes in the TCGA-training set (Supplementary Table S2). Subsequently, multivariate Cox regression and stepwise regression models were employed for identifying the prognostic signature. A set of six MDSC-related genes (*ALDOA*, *CD52*, *FTH1*, *RTN4*, *SLC2A3*, and *TNFAIP6*) were finally trained as the prognostic signature (Figure 4A) (Materials and methods). Further analyzing the expression levels of the signature genes, we found that *TNFAIP6* (TNF alpha induced protein 6) was an MDSC-specific marker among myeloid subpopulations (Figure 4B; Supplementary Table S3). In addition, we employed a risk-scoring model based on the MDSC prognostic signature. The risk score of each patient could be expressed as Risk score = (0.0026681*ALDOA) + (−0.0126845*CD52) + (0.0015119*FTH1) + (0.0125243*RTN4) + (0.0154432*SLC2A3) + (0.0052426*TNFAIP6). We next subgrouped patients into high- and low-risk groups based on the median value of risk scores. Compared to the low-risk group, patients in the high-risk group showed worse prognosis outcomes in the TCGA training set, TCGA test set, and two external test datasets (GSE65858 and GSE41613) (Figure 4C). Moreover, we also evaluated the capacity of the MDSC risk group as the independent prognostic predictor. By adjusting the effects of tumor stage, age, and gender, the risk group is still a robust poor prognostic signature (Figure 4D). These results showed that the MDSC-related genes exhibited outstanding performance in HNSC prognostic prediction.

### Intercellular communication associated with MDSCs in HNSC

**FIGURE 5:**
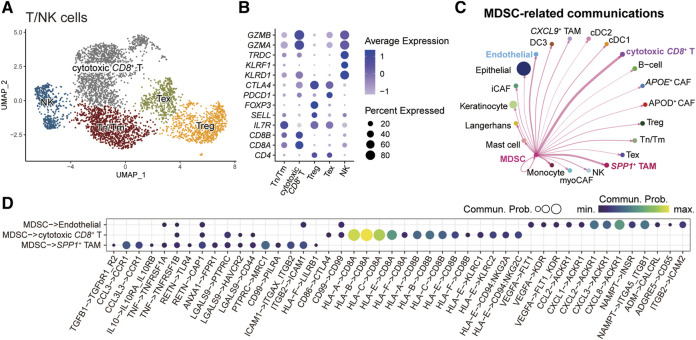
Intercellular communications between MDSCs and other microenvironment cells. **(A)** UMAP plot of T/NK subpopulations, dots colored by different cell types. **(B)** Dot plot showing the expression level of cell-type-specific gene markers. **(C)** Circle plots displaying the inferred network of MDSC-related cell communications. Edge width is proportional to the inferred communication counts. **(D)** Dot plot showing the ligand-receptor pairs from MDSC to endothelial cell, cytotoxic *CD8* ^+^ T cell, and *SPP1* ^+^ TAM. Dots are sized by the inferred communication probabilities.

We next characterized the role of MDSC in the tumor microenvironment by dissecting intercellular communications. We first subgrouped the T/NK cells (Figure 5A) and fibroblasts (Supplementary Figure S4). For T/NK cells, we identified five subpopulations and annotated them by the known gene markers: naïve/memory T-cells (Tn/Tm) (*IL7R*, *SELL*), cytotoxic *CD8* ^+^ T-cells (*CD8A*, *CD8B*, *GZMA*, *GZMB*), regulatory T-cells (Treg) (*FOXP3*), exhausted T-cells (Tex) (*PDCD1*, *CTLA4*), and natural killer cells (NK) (*KLRF1*, *KLRD1*, *TRDC*) (Figure 5B). For fibroblasts, we recognized five subgroups, including *APOE* ^+^ cancer-associated fibroblasts (CAFs), *APOD* ^+^ CAFs, myofibroblastic CAFs (myoCAFs), inflammatory CAFs (iCAFs), and proliferating cells (Supplementary Figure S4). Subsequently, we employed CellChat to explore the crosstalk between MDSCs and other microenvironment cells (Figure 5C). The result showed that MDSC mainly communicated with cytotoxic *CD8* ^+^ T-cells, *SPP1* ^+^ TAMs, and endothelial cells. Further parsing of the ligand-receptor (LR) pairs between MDSC and its three partners, we found that several non-classical MHC class I molecules, including *HLA-E* and *HLA-F*, mediated the communications from MDSC to cytotoxic *CD8* ^+^ T-cells (Figure 5D), which was important for shaping immunosuppressive microenvironment [38, 39]. In addition, MDSC could interplay with *SPP1* ^+^ TAMs via the LR pair *CCL3/CCL3L3-CCR1* (Figure 5D), which might facilitate the recruitment of *SPP1* ^+^ TAMs in tumor tissue [40, 41]. We also observed that MDSC-secreted *VEGFA* and multiple chemokines *CCL2*, *CXCL1*, *CXCL2*, *CXCL3*, and *CXCL8* could act on endothelial cells via *VEGFA-KDR*/*FLT1* and *CCL2/CXCLs-ACKR1* interactions, which were crucial for tumor angiogenesis [42, 43]. We also evaluated the clinical implications of MDSC-related LR pairs by analyzing their associations with the probabilities of patients’ OS and progression-free survival (PFS) (Supplementary Figure S5). We found some immunosuppressive (*CD86-CTLA4*, *HLA-E-CD8A/CD94_NKG2C/CD94_NKG2A, HLA-F-CD8A*) and angiogenesis-related (*CXCL1-ACKR1*) LR pairs showed a significant association with patients’ OS and PFS. These results highlight the potential of MDSC in shaping the immunosuppressive microenvironment and promoting tumor angiogenesis, which is also associated with tumor development and progression.

## Discussion

Head and neck squamous cell carcinoma (HNSC) is one of the most common malignancies in the head and neck region. In this study, scRNA-seq data was used to dissect the landscape of the tumor microenvironment of HNSC. We found that there were nine major cell types, including epithelial cells, keratinocytes, fibroblasts, endothelial cells, myeloid cells, T/NK cells, mast cells, B cells, and proliferating cells. Among these, myeloid cells play a critical role in regulating the immune response in the HNSC tumor microenvironment. We further analyzed the subpopulations of myeloid cells, including dendritic cells (DCs), tumor-associated macrophages (TAMs), monocytes, myeloid-derived suppressor cells (MDSCs), Langerhans cells, and proliferating cells. We investigated the proportions of myeloid subpopulations among HNSC patients and found that *SPP1* ^+^ TAM and MDSC had higher fractions compared to other myeloid cells. We also found that highly-infiltrated MDSCs prompted poor prognostic risks, suggesting that MDSC infiltration was a prognostic risk factor. Recent studies also demonstrated that the infiltration levels of MDSC were increased in the HNSC tumor tissues, and their presence was positively associated with advanced T stage, higher pathological grade, lymph node metastasis, and poor prognosis [16]. Furthermore, in a co-culture system, tumor-related MDSCs were found to promote the progression of HNSC by enhancing cell proliferation, migration, epithelial-mesenchymal transition (EMT), and vasculogenic mimicry (VM). These findings suggest a reciprocal interaction between MDSCs and tumor cells, facilitating the malignant progression of HNSC and enhancing the immunosuppressive properties of MDSCs.

To better apply MDSCs in survival prediction, we explored the prognosis values of MDSC-related genes. We identified a set of six MDSC-related genes that were trained as the prognostic signature, including *ALDOA*, *CD52*, *FTH1*, *RTN4*, *SLC2A3*, and *TNFAIP6*. We also employed a risk-scoring model based on the MDSC prognostic signature, and patients were subgrouped into high- and low-risk groups based on the median value of risk scores. The high-risk group showed worse prognosis outcomes in the TCGA training set, TCGA test set and two external test datasets, suggesting that the MDSC risk score could be a useful independent prognostic predictor.

Further analysis of MDSC-related cell communications, we found MDSC showed the potential to recruit *SPP1* ^+^ TAM, inhibit the activity of cytotoxic T cells, and promote endothelial outgrowth, which was associated with immunosuppressive TME. In addition to MDSC, *SPP1* ^+^ TAM also played an important role in shaping the immunosuppressive TME [44]. Notably, the prognostic signature also showed an association with hypoxic immunosuppressive TME. In addition to *TNFAIP6*, another five exhibited a general expression across myeloid subtypes (Figure 4B). These genes may also be regulated by the immunosuppressive microenvironment [45–47], and their association with MDSCs will be investigated in our future study. For instance, *ALDOA* and *SLC2A3* were found to be linked to glycolysis, indicating their potential role in energy metabolism [48–50]; *RTN4* was identified as a possible contributor to tumor angiogenesis, suggesting its involvement in the formation of new blood vessels to support tumor growth [51]; *TNFAIP6* emerged as a significant regulator of extracellular matrix organization, implying its influence on the structural integrity and composition of the tumor microenvironment [52–58].

Overall, this study provides valuable insights into the landscape of the tumor microenvironment of HNSC and highlights the critical role of myeloid cells in regulating the immune response. The findings also suggest that MDSC infiltration is a prognostic risk factor in HNSC patients and that the MDSC prognostic signature could be a useful tool for predicting patient outcomes. These results may have important implications for the development of novel immunotherapeutic strategies for HNSC.

## Data Availability

All the data used in this study could be accessed freely in GEO and TCGA.
