# Supplementary material for: Single-cell transcriptome analysis reveals the clinical implications of myeloid-derived suppressor cells in head and neck squamous cell carcinoma
Source: Pathol Oncol Res. 2023 Jul 5;29:1611210. doi: 10.3389/pore.2023.1611210 (PMC10354270; doi:10.3389/pore.2023.1611210)
Supplement: Supplementary file 1 [file DataSheet2.docx]

**Single-cell transcriptome analysis reveals the clinical implications of myeloid-derived suppressor cells in oral squamous cell carcinoma**

Jiang *et al.*


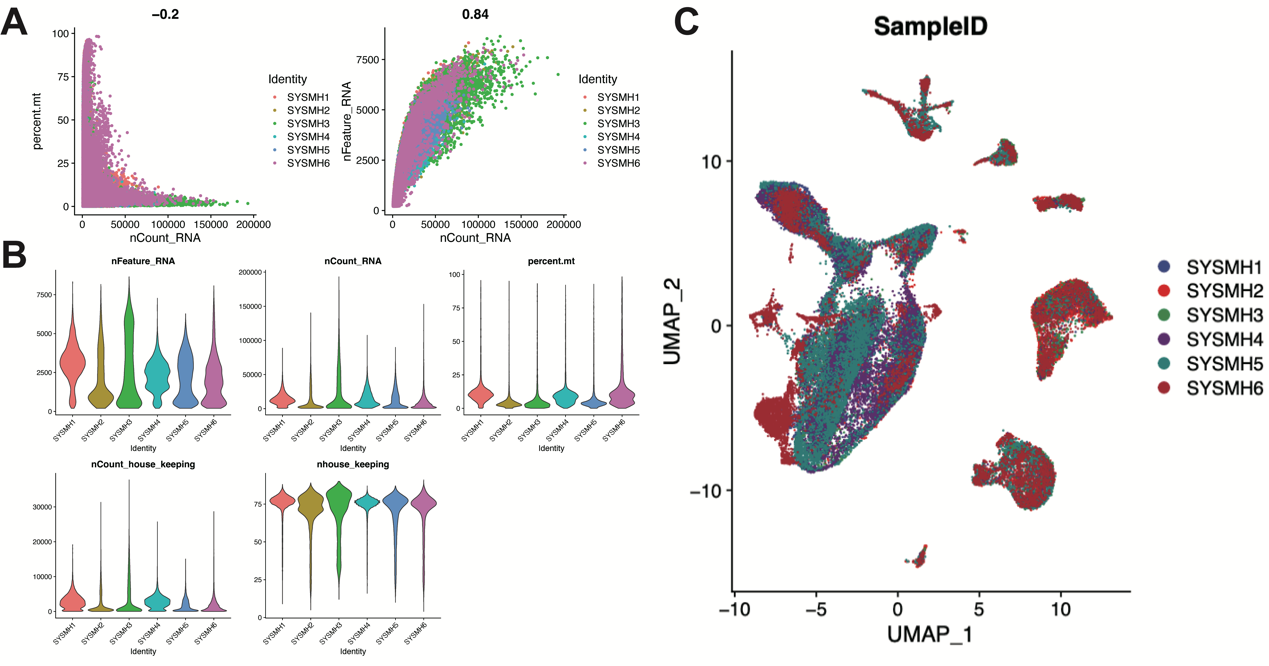


**Figure S1. Quality control of single-cell data.** A. Scatter plot showing the correlation between sequencing depth and mitochondrial gene. B. Violin plot showing the number of genes and count, the percent of mitochondrial genes, and housekeeping genes detected in each cell. The x-axis represents the patient, and the y-axis represents the number of genes. C. UMAP plot colored by samples.


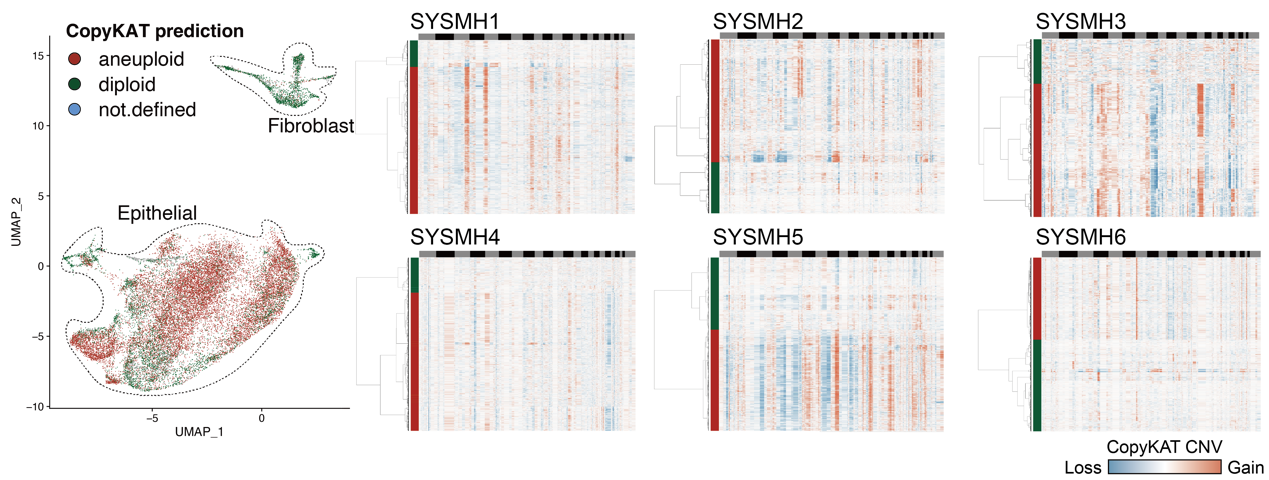


**Figure S2. Identification of OSCC tumor Cells by CopyKAT with fibroblast as a negative control.** UMAP plot showing the aneuploid/diploid identified by CopyKAT. Clustered heatmap showing the copy number profiles estimated from scRNA-seq data of each donor using CopyKAT.


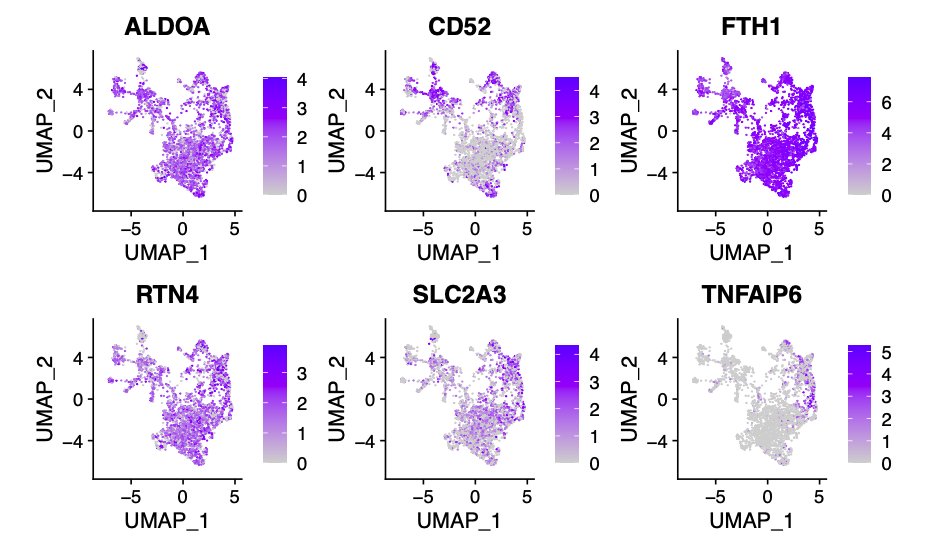


**Figure S3. Expression levels of MDSC-related prognostic markers.**


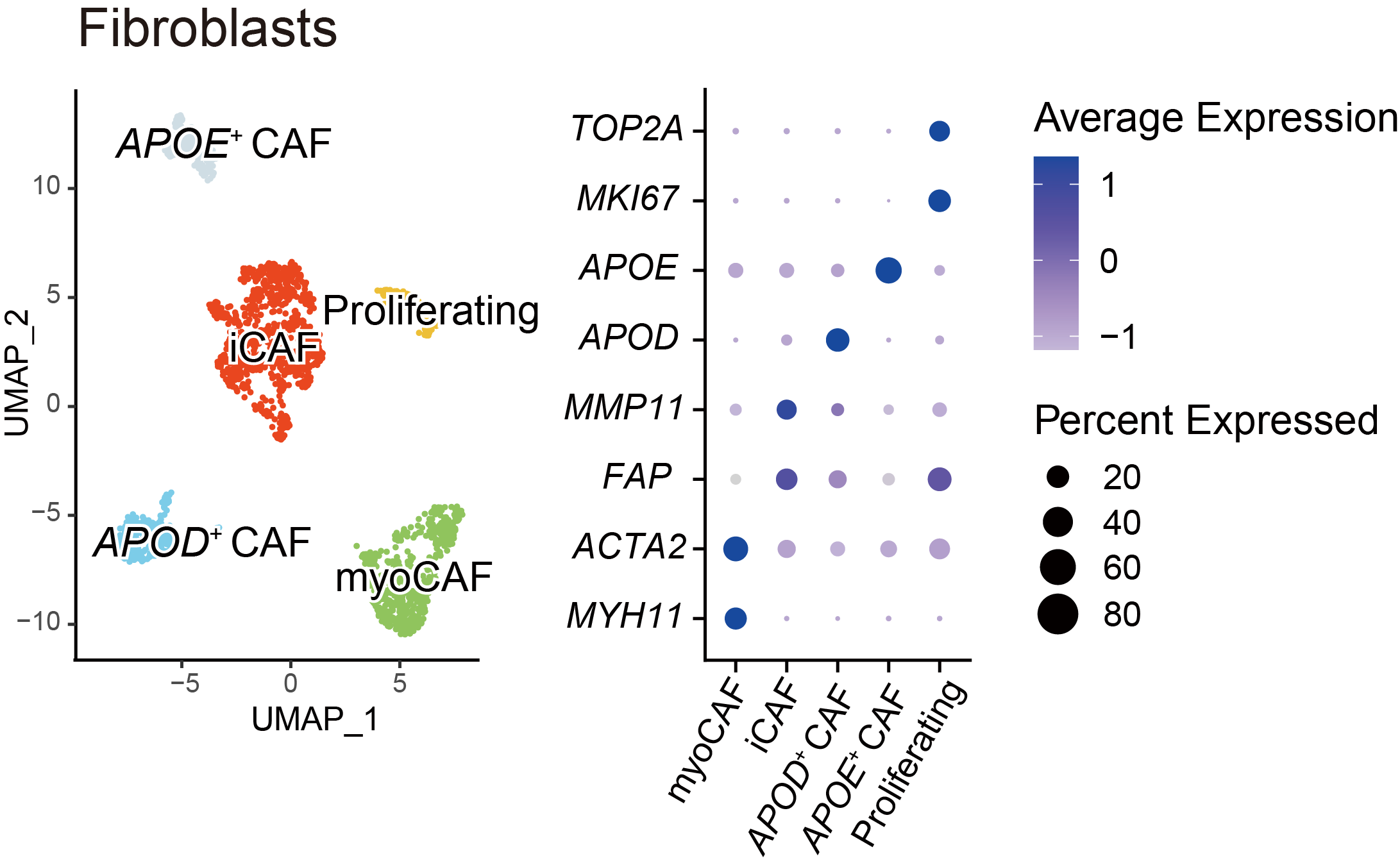


**Figure S4. Subpopulations of fibroblasts.** UMAP plot of fibroblast subpopulations, dots colored by different cell types. Dot plot showing the expression level of cell-type-specific gene markers.


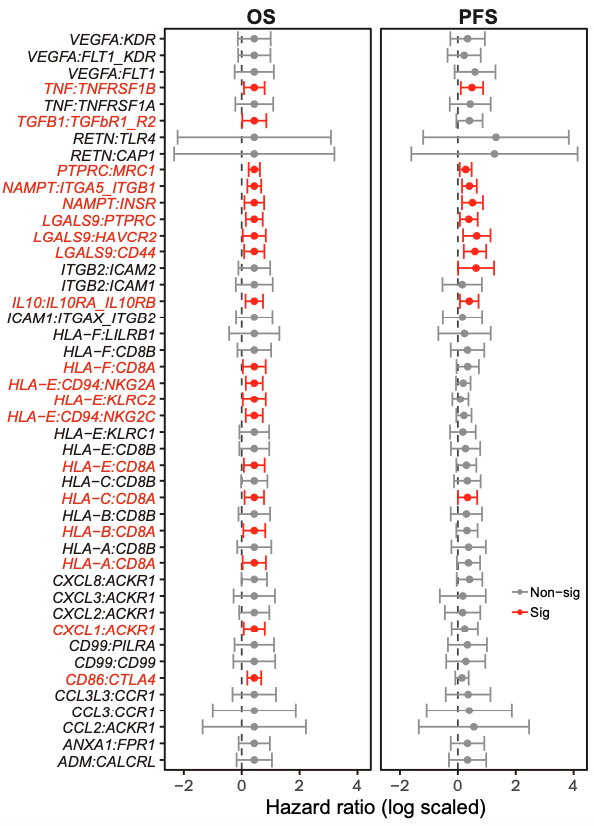


**Figure S5. Forest plot showing the hazard ratio of risk scores constructed by the expression levels of MDSC-related LRs in the TCGA cohort.** We construct the multivariate Cox regression model using each MDSC-related LRs and calculate the risk score, respectively. Subsequently, the univariate Cox regression model was employed to evaluate the significance between the LR risk score and the patient’s overall survival (OS) and progression-free survival (PFS).
